# Brain and eyes of *Kerygmachela* reveal protocerebral ancestry of the panarthropod head

**DOI:** 10.1038/s41467-018-03464-w

**Published:** 2018-03-09

**Authors:** Tae-Yoon S. Park, Ji-Hoon Kihm, Jusun Woo, Changkun Park, Won Young Lee, M. Paul Smith, David A. T. Harper, Fletcher Young, Arne T. Nielsen, Jakob Vinther

**Affiliations:** 1 Division of Polar Earth-System Sciences, Korea Polar Research Institute, 26 Songdomirae-ro, Yeonsu-gu, Incheon, 21990 Republic of Korea; 2 Polar Science, University of Science and Technology, 217 Gajeong-ro, Daejeon, 34113 Republic of Korea; 3 Division of Polar Life Sciences, Korea Polar Research Institute, 26 Songdomirae-ro, Yeonsu-gu, Incheon, 21990 Republic of Korea; 4 Oxford University Museum of Natural History, Parks Road, Oxford, OX1 3PW UK; 5 Palaeoecosystems Group, Department of Earth Sciences, Durham University, Durham, DH1 3LE UK; 6 Schools of Earth Sciences and Biological Sciences, University of Bristol, Woodland Road, Bristol, BS8 1UG UK; 7 Department of Geoscience and Natural Resource Management, University of Copenhagen, Øster Voldgade 10, Copenhagen, DK-1350 Kbh K Denmark

## Abstract

Recent discoveries of fossil nervous tissue in Cambrian fossils have allowed researchers to trace the origin and evolution of the complex arthropod head and brain based on stem groups close to the origin of the clade, rather than on extant, highly derived members. Here we show that *Kerygmachela* from Sirius Passet, North Greenland, a primitive stem-group euarthropod, exhibits a diminutive (protocerebral) brain that innervates both the eyes and frontal appendages. It has been surmised, based on developmental evidence, that the ancestor of vertebrates and arthropods had a tripartite brain, which is refuted by the fossil evidence presented here. Furthermore, based on the discovery of eyes in *Kerygmachela*, we suggest that the complex compound eyes in arthropods evolved from simple ocelli, present in onychophorans and tardigrades, rather than through the incorporation of a set of modified limbs.

## Abstract (web summary)

The arthropod head is complex and its evolution has been difficult to reconstruct. Here, Park et al. describe new specimens of the Cambrian stem-group euarthropod *Kerygmachela* that preserve evidence of primitive compound eyes and a unipartite brain, providing insight into the structure of the early arthropod head.

## Introduction

Resolving the evolutionary origin of the frontal appendages and eyes of stem-group euarthropods is crucial for elucidating the segmental affinity of the head of arthropods^1,2^. The arthropod brain is composed of three distinct segments (tritocerebral) that innervate corresponding appendages. The affinity and homology of arthropod appendages is essential to the identification of which appendage is innervated by which brain segment. Among these, the origin of the labrum has been much debated^3–5^. The recent discovery of an anomalocaridid with the brain preserved suggests a protocerebral innervation of the frontal appendages in stem-group euarthropods, and a homology between the frontal appendage and the labrum^1^; however, this interpretation remains contested^6,7^. Furthermore, it has recently been proposed that a subdivision of the protocerebrum into prosocerebrum and archicerebrum occurred through the fusion of two appendage-bearing segments, in which the eyes are modified appendages of the archicerebral segment^2,3,8^.

*Kerygmachela kierkegaardi*^9^ is known only from the Buen Formation (Cambrian Stage 3) of Sirius Passet, North Greenland^10^. This relatively rare, gilled lobopod exhibits some anomalocaridid-like features, such as the trunk “gills”^9,10^, diverticulae^11^, and its frontal appendages^9,10^. Nevertheless, its overall anatomy is more plesiomorphic, lacking clear arthrodization/arthropodization and instead exhibiting a softer, annulated external cuticula comparable to onychophorans and tardigrades along with the diverse host of Cambrian lobopodians. Therefore, this taxon may provide crucial evidence for understanding early character evolution in the panarthropod head. To date, knowledge of its morphology has been based on specimens collected from weathered screes. However, for this study, unweathered fossils of *Kerygmachela* were collected from the rock exposure of the Buen Formation in Sirius Passet, and these specimens include structures preserved as carbonaceous reflective films in the head. These structures conform to eyes and nervous tissue in their anatomy and preservation mode. The findings shed light on the ancestral condition of the panarthropod brain and the origin of complex arthropod compound eyes. The new material, furthermore, provides novel information on the overall anatomy of *Kerygmachela*.

## Results

### Head region

**Fig. 1:**
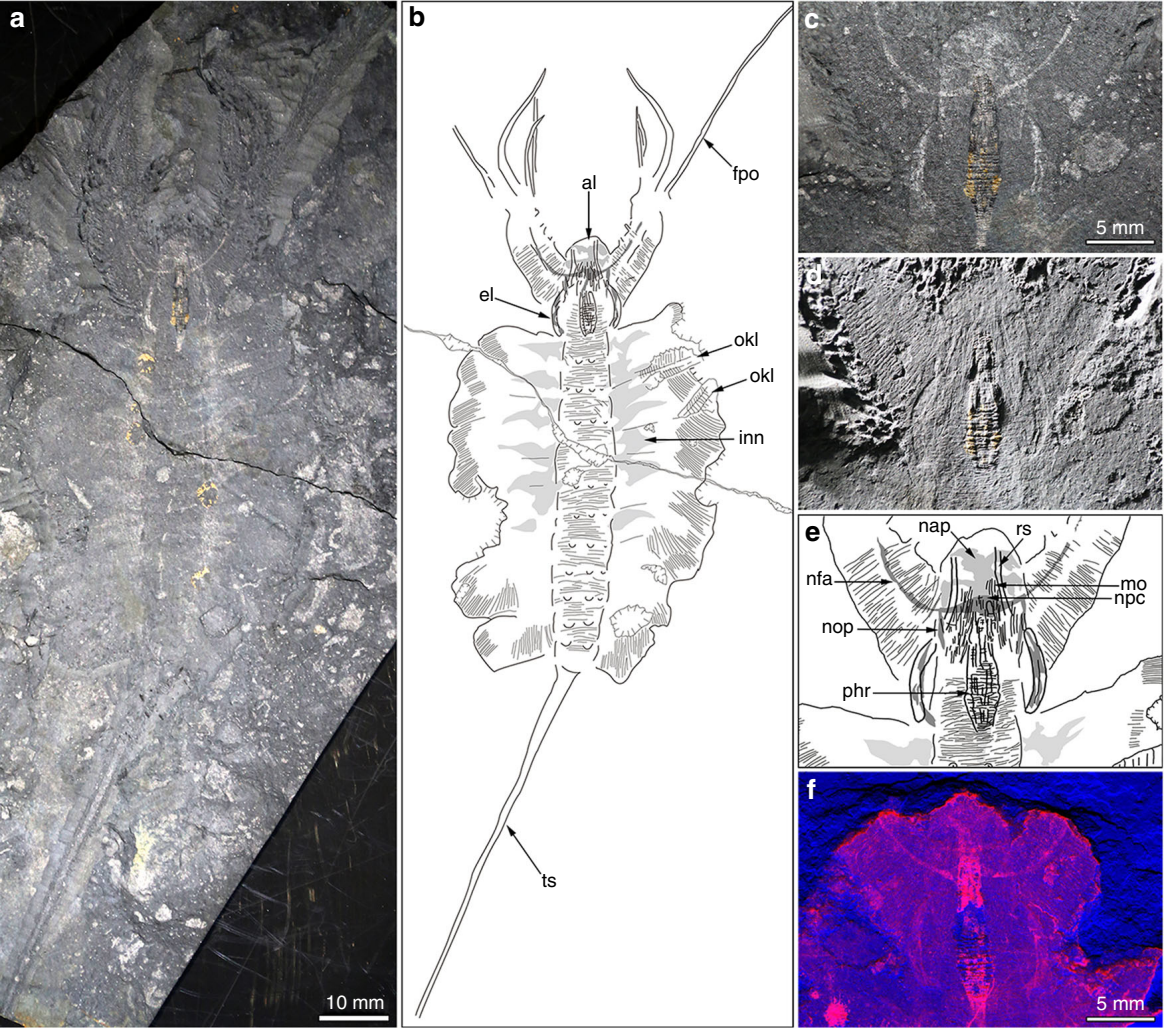
*Kerygmachela kierkegaardi* from the Cambrian Stage 3 of North Greenland. **a–e** MGUH 32048a. **a** Overview of part. **b** An interpretive drawing of **a**. **c** and **d** Magnified images of the head region of **a**. **c** Under high-angle polarized lighting. **d** Under low-angle lighting. **e** Interpretive drawing of **c** and **d**. **f **MGUH 32048b, the counterpart of MGUH 32048a; a wavelength dispersive X-ray elemental map of carbon of the head region. Carbon-rich region (red), superimposed upon the topographic map (blue), representing the frontal appendage nervous tracts and the anterior neural projections. al anterior lobe, el eye lobe, fpo outermost frontal process, inn non-neural impression, mo mouth opening, nap anterior neural projection, nfa frontal appendage nervous tract, npc protocerebrum, nop optic neuron, okl overprint of *Kleptothule* arthropod specimen, phr pharynx, rs rostral spine, ts tail spine

**Fig. 2:**
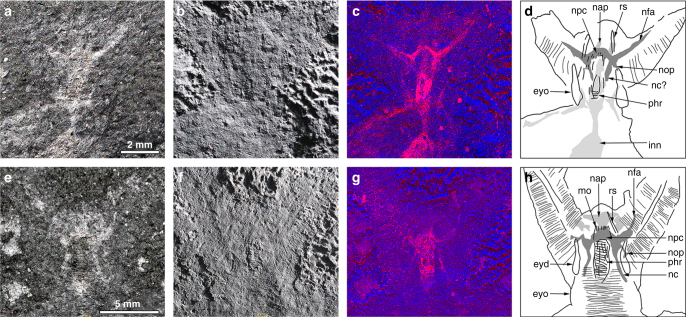
Details of head structure in *Kerygmachela kierkegaardi*. **a–d** MGUH 32049. **a** Under high-angle polarized lighting. **b** Under low-angle lighting. **c** Wavelength dispersive X-ray elemental map of carbon of the head region. **d** Interpretive drawing of **a** and **b**. **e–g** MGUH 32050. **e** Under high-angle polarized lighting. **f** Under low-angle lighting. **g** Wavelength dispersive X-ray elemental map of carbon of the head region. **h** Interpretive drawing of **e** and **f**. eyd displaced eye structure, eyo original outline of eye lobe, inn non-neural impression, nap anterior neural projection, nc nerve cord, nfa frontal appendage nervous tract, npc protocerebrum, nop optic nerve, phr pharynx, rs rostral spine

**Fig. 3:**
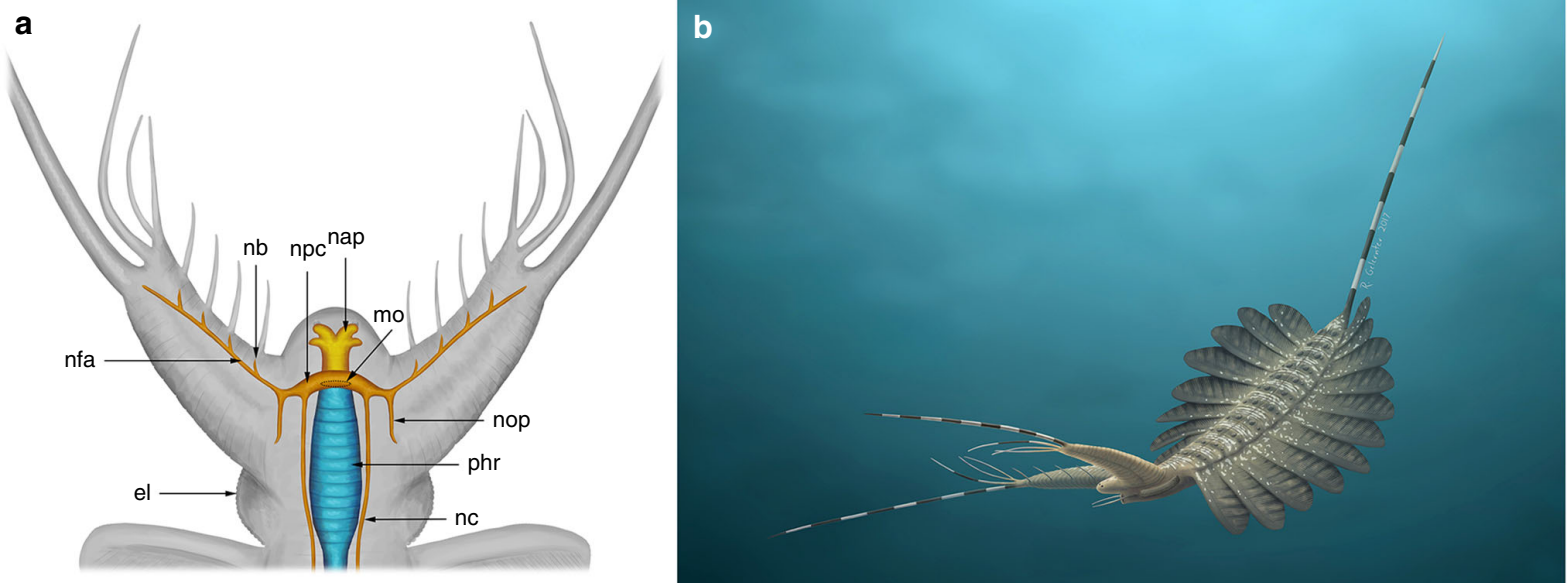
Reconstruction of *Kerygmachela kierkegaardi*. **a** Dorsal reconstruction of the head region with the central nervous system (orange), anterior neural projection (yellow), and muscular pharynx (blue). **b** Artistic reconstruction of *K*. *kierkegaardi*. el eye lobe, mo mouth opening, nap anterior neural projection, nb branching of nerve, nc nerve cord, nfa frontal appendage nervous tract, npc protocerebrum, nop optic nerve, phr pharynx. Artwork by Rebecca Gelernter (nearbirdstudios.com)

The newly collected material described here comprises 15 specimens of *Kerygmachela*. The head forms a rounded anterior lobe extending between the frontal appendages (Fig. 1a–e; Supplementary Fig. 1a–c). Accordingly, the position of the anteriorly facing mouth is not terminal^10^, but is situated ventrally below the anterior lobe, between a pair of structures termed as rostral spines^10^ (Figs. 1e, 2h; Supplementary Fig. 1c). At the base of the frontal appendages are structures interpreted as eyes, based on both their position and preservation, with distinctive relief and high reflectivity similar to the preservation of eyes in arthropods from the Burgess Shale (see Fig. 2 in ref.^12^). Their outline is reminiscent of the kidney- or sickle-shaped eye lobes of trilobites (Figs. 1c–e, 3a, Supplementary Figs 2b–d, 8a–e). We interpret the eyes as situated ventrolaterally, because they are best delineated in ventral specimens where mouth structures and rostral spines are most visible (Fig. 1d,e; Supplementary Figs 1a–c, 2, 8a–e). In dorsal specimens, the epidermal annulations continue over the eye lobes (Supplementary Fig. 1f, i). The anterior half of the eye is located under the posterior part of the frontal appendages (Fig. 1d,e; Supplementary Figs 2c–d, 8a–e). The presence of relatively marked relief in the eyes (Fig. 1d; Supplementary Figs 2b, 8b,d) contrasts with Cambrian lobopodians that show a single or a few clustered visual units lacking such elevation^13,14^. This indicates that there was a visual surface formed by the contiguous arrangement of visual units, suggesting that the visual structures seen in *K*. *kierkegaardi* represent primitive compound eyes, further corroborated by their large size. Their morphology does not match camera eyes, which are globular. The irregular shape of the eye lobes in other specimens (Fig. 2d,h; Supplementary Fig. 1c, f, i) suggests that they have been subject to some post-mortem decay. The ventro-lateral position of the eyes in *Kerygmachela* is remarkable, but not unique; the ocellus-like eye of the Cambrian lobopodian *Onychodictyon ferox* was also situated ventral to the antennae^15^. A pair of rostral spines flank the mouth, attached just behind the mouth opening in MGUH 32048b (Fig. 1e). Sub-circular structures are present at the base, which are likely to be apodemes (Supplementary Fig. 1c).

We interpret the anterior reflective patches, lacking any relief and located between the frontal appendages as a centralized nervous system (Figs. 1a,c, 2a,e; Supplementary Figs 1a, d, g). These structures are similar to the nervous tissue described in the anomalocaridid *Lyrarapax unguispinus*^1^ (Fig. 3a) and conform to nervous tissue in their topology when compared with living relatives. The nervous system is usually best observed when mapping carbon distribution by wavelenth dispersive X-ray spectrometry (WDS) (Figs. 1f, 2c,g; Supplementary Figs 2, 4–6). MGUH 32049 (Fig. 2c) appears to be the best preserved, reconciling evidence from the range of fossils available in which some appear to be more decayed before taphonomic stabilization (Fig. 2g; Supplementary Fig. 5d). The brain shape in this specimen appears to be reminiscent of the anteriorly rounded central brain neuropil in a eutardigrade embryo^16^ (Supplementary Fig. 10a, b). If the prominent carbon-rich area in the WDS carbon map represents the central brain neuropil, the surrounding reflective area of the brain may be the trace of brain cells as seen in tardigrades^16^. The taphonomic pathways leading to nervous tissue preservation are still not fully understood. It has been noted that nervous tissue decays rapidly^17^, but the relative pace at which tissues decay is a poor proxy for their preservation potential^18^. Certain lipids are common organic biomarkers due to their stable molecular structure, and nervous tissues are lined by lipid-rich tissues, which may explain their preservation potential^19,20^.

The mouth opening, at the anterior end of a pharynx that is usually mineralized in the fossils, could lie just posterior to the brain (Supplementary Fig. 1a–c), but in the largest specimen, the anterior margin of the mineralized pharynx is situated anterior to the brain area (Fig. 1c–e), which might be due to decay deformation. Unlike *L*. *unguispinus*, simple nervous tracts without an associated ganglion are supplied from the lateral margin of the brain to innervate the two frontal appendages (Fig. 3a). The apparent bifurcation of the nervous tract for the frontal appendages observed in MGUH 32053 probably supplied a nerve to an anterior spine (Supplementary Fig. 1g, i). The nervous tract extends almost to the tip of the frontal appendage, and likely innervates the outermost and longest frontal process (Supplementary Fig. 1a, c; Supplementary Fig. 3). There are anterior neural projections from the brain into the anterior lobe (Figs. 1a–e, 2, 3a; Supplementary Fig. 1). The relative size of the anterior neural projection tends to be more developed in larger specimens (Fig. 2; Supplementary Fig. 1). Less conspicuous optic nerves root at the lateral margin of the brain, indicating that the brain is protocerebral (Figs. 1c,e, 2a,e,d,h, 3a; Supplementary Fig. 1a, c, d, f, g, i). A pair of nerve cords (or connecting cords^21^) is tentatively present in MGUH 32053 and MGUH 32050 (Fig. 2d,h; Supplementary Fig. 1g, i). In more derived arthropods, the deutocerebrum innervates the second anteriormost appendages. Due to its morphology and since the first post-protocerebral appendage is situated far behind the protocerebrum, it can be concluded that the brain of *Kerygmachela* is unipartite.

### Trunk region

The trunk had 11 pairs of flaps giving it an oval outline with a single, unsegmented tail spine. Eleven rows of tubercles are present in the axial region, corresponding to each set of lateral flaps, with four tubercles present in each row. The tubercles become more prominent posteriorly. The central two tubercles are larger than the outer ones and become more widely spaced posteriorly, eventually becoming almost superimposed on the lateral ones in the distal region (Fig. 3b; Supplementary Figs 5a–c, 6a–c, 8a–c, f–h, 9a–e). The lateral flaps appear successively smaller rearward in the holotype^10^, but this is due to their backwards-facing aspect in this specimen. In the new material, the fourth and fifth pair of lateral flaps appear to be largest, rendering an oval outline in dorsal view (Figs. 1a,b, 3b; Supplementary Fig. 6a–c). Although the holotype of *Kerygmachela* has a single long tail spine, the animal was interpreted to have a pair of tail spines^9,10^. However, all new specimens in this study show a single, long, medially attached, and unsegmented tail spine, when preserved (Fig. 1a,b; Supplementary Figs 6a–c, 7, 8a–c, 9). The tail spine is almost as long as the body, excluding the frontal appendages. There are derived stem-group euarthropods with a single tail spine-like structure which might be homologous; *Schinderhannes bartelsi* from the Devonian Hunsrück Slate has a long terminal spine with a median keel^22^, and *Anomalocaris canadensis* from the Burgess Shale has an elongated “blade”^23^.

## Discussion

The segmental origin of frontal appendages in stem-group euarthropods is one of the key issues for solving the “arthropod head problem”^4^, and identifying their innervation has been regarded as a possible solution to this problem^5^. The frontal appendages of stem-group euarthropods have been interpreted as innervated from the protocerebrum, being homologous to the labrum^1,3,4^, or the deutocerebrum, being homologous to antennule or chelicera^5,24,25^. The frontal appendage nerves of the anomalocaridid *L. unguispinus* have been interpreted as being supplied from the protocerebrum, based on the pre-ocular position of the innervation^1^. It has been argued, however, that the relative position of the eyes and frontal appendages does not necessarily reveal their segmental identity in dorso-ventrally compressed fossils^6^, since in euarthropod development, deutocerebral appendages may become displaced anteriorly^3^. The presence of a unipartite, and hence protocerebral, brain in the stem-euarthropod *Kerygmachela*, substantiates the suggested homology between the stem-euarthropod frontal appendages and the labrum of euarthropods^1,3,4^.

In contrast to *L*. *unguispinus*^1^, the more basal *K*. *kierkegaardi* does not show any trace of ganglia within the frontal appendages (Figs. 1, 2, 3a; Supplementary Fig. 1). This indicates that the pre-protocerebral ganglion of *L*. *unguispinus* may have been an autapomorphic feature, and that the frontal appendages are not pre-protocerebral in origin.

Developmental studies have found a genetic subdivision of the arthropod protocerebrum—*six3*/*optix* is expressed in the anteromedian area of the protocerebrum, while *otx*/*otd* is expressed in the lateral regions^26,27^. This has led to the suggestion that the ancestral arthropod brain was bipartite with a prosocerebrum innervating the euarthropod labrum or the onychophoran antennae, while the posterior archicerebrum originated from the incorporation of a limb pair, modified into stalked eyes seen primitively in euarthropods and their proximal stem groups, the radiodontans^2,3,8^.

**Fig. 4:**
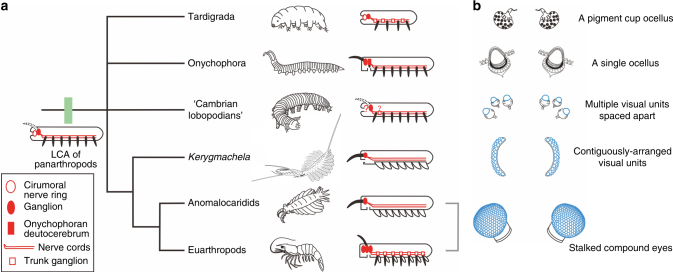
A panarthropod phylogenetic tree with diagrammatic summary of central nervous system (CNS) and evolution of visual structures within Panarthropoda. **a** Diagrammatic representation of the CNS in panarthropod lineages, with the inferred CNS condition in the last common ancestor (LCA) of panarthropods. Although it is not unequivocal, the lack of segmental ganglia in the trunk of onychophorans^7^ and the stem-group euarthropod *Lyrarapax*^1^ suggests that segmental ganglia were absent in the LCA of panarthropods. **b** Visual structures of different panarthropod lineages. The diagrammatic representations of the visual organs of Tardigrada and Onychophora are modified from ref.^13^. “Cambrian lobopodians” is also referred to as xenusians in the literature^37^. The polytomy in this cladogram represents the unresolved phylogenetic relationship among Tardigrada, Onychophora, “Cambrian lobopodians,” and the total-group euarthropods

The plesiomorphic condition of visual structures in panarthropods has remained elusive due to the currently ambiguous phylogenetic relationships of the Tardigrada, Onychophora, and Cambrian lobopodians. Nevertheless, it can now be suggested that the single or spaced-apart multiple sessile visual units of the “Cambrian lobopodian” grades^13^ evolved into the compound eyes of *Kerygmachela*, from which the stalked compound eyes of anomalocaridids^28^ and euarthropods^2^ arose (Fig. 4b).

Recognition of rather large-sickle-shaped eyes in *Kerygmachela* contradicts the suggestion that the small, circular structures at the root of the rostral spines represent eyes^10^. Rather, we draw attention to the architectural resemblance between the buccal apparatus of tardigrades and the rostral spines–pharynx complex of *Kerygmachela*, which will require further scrutiny (Supplementary Fig. 10c, d). If the tardigrade stylets are truly homologous to the rostral spines of *Kerygmachela*, it would cast doubt on the suggested protocerebral appendage origin of the tardigrade stylets^29^.

The position of the anterior lobe in *Kerygmachela* is reminiscent of the anterior sclerite in more derived arthropods, which is a non-appendicular exoskeletal structure containing protocerebral frontal organs^12^, which may have arisen from a less sclerotized structure as observed in *Kerygmachela*.

The frontal organs have been regarded as possible median eyes^12,30^. The anterior neural projections in the anterior lobe of *Kerygmachela* may have supplied neurons to possible dorsal median visual units, and thus the dorsal median visual units could have been used for anterodorsal vision supplementing the ventrolaterally positioned compound eyes.

The unipartite brain of *Kerygmachela* may thus prove essential for inferring the brain condition of the last common ancestor (LCA) of panarthropods. Although brain anatomy is hard to distinguish even in extant animals, recent studies suggest that tardigrades have a unipartite (protocerebral) brain^16,29,31^. The onychophoran brain has been considered to be bipartite^32^, but derived^20^. The presence of a unipartite brain, consisting of a protocerebrum in the stem-group euarthropod *Kerygmachela*, therefore, suggests that it represents the primitive panarthropod condition along with tardigrades. In addition, recent paleontological evidence indicates that there are close architectural similarities in circumoral structures between other ecdysozoans (cycloneuralians) and basal panarthropods^33,34^. Since all cycloneuralians have a collar-shaped circumoral nerve ring with anterior and posterior rings of neuronal somata, it is likely that the LCA of panarthropods had a circumoral nerve ring, along with a protocerebum (Fig. 4a). In this regard, the presence of a nerve ring in a eutardigrade with an anteriorly facing terminal mouth is particularly significant^31^ (Supplementary Fig. 10f), which are absent in heterotardigrades (Supplementary Fig. 10g). Indeed, peribuccal lamellae, and buccopharyngeal teeth (mucrones) in the eutardigrade mouth (Supplementary Fig. 10e) are morphologically and topologically comparable to the conical spines surrounding the mouth, and pharyngeal teeth of priapulid-like cycloneuralians, respectively.

Inference of an ancestral condition based on living animals is often hampered by convergent evolution among distant lineages, representing parallel adaptations to changes in the biosphere and evolutionary escalation. The discovery of the simple unipartite brain in stem-group euarthropods corroborates the ancestral simplicity of the panarthropod brain, and also suggests that the complex neural concentrations, such as tripartite brains^35,36^ in euarthropods and chordates, are the result of convergent adaptations.

## Methods

### Material studied

Fifteen specimens of *K. kierkegaardi* were collected in situ from the Buen Formation at the main locality in Sirius Passet, Nansen Land, North Greenland during the expeditions in 2011, 2016, and 2017. The specimens are preserved as thin reflective films, as in documented fossils of the other animals^38–40^. The specimens are deposited in the Geological Museum, Natural History Museum of Denmark, University of Copenhagen, prefixed with MGUH (MGUH 32048–32062). Fossils were mechanically prepared using an AUTOMEL Electric Engraver of Dong Yang Electric Co.

### Image acquisition

The fossils were photographed in the laboratory and submerged in ethanol, with a Canon EOS 6D using a Canon EF 100 mm f/2.8 L IS USM macro lens equipped with a polarizing filter (ELVA XS-Pro1 Digital CIR-PL 67 mm). High-angle polarized lighting was used to obtain the maximal reflective image of the specimens. Low-angle (close to horizontal) lighting was also used from various orientations to enhance recognition of low relief in the fossils. Images were cropped, adjusted, and enhanced by means of brightness and contrast in Adobe Photoshop CS6. The raw images for polynomial texture mapping were acquired by a self-crafted system permitting lighting from 50 different directions and a Canon EOS 60D equipped with a Canon EF 100 mm f/2.8 USM macro lens. The 50 images were taken for each white-coated specimen, which were together converted into a PTM format file. The PTM file was then run in RTI Viewer software which is freely downloadable at http://culturalheritageimaging.org/What_We_Offer/Downloads/, in order to enhance surface details of the fossils.

### X-ray elemental mapping

Fossils were gold coated using a Cressington 108 Auto sputter coater with 40 mA for 60 s. X-ray elemental maps for C, Na, Mg, Al, P, S, Cl, K, Ca, and Fe were obtained using the JEOL JXA-8530F field emission electron microprobe at the Korea Polar Research Institute, equipped with five wavelength dispersive X-ray spectrometers (WDS). We employed stage mapping with an acceleration voltage of 20 kV, beam current of 100 nA, beam size of 5 μm, dwell time of 10–20 ms, and step size of 10–25 μm. Secondary electron (SE) and backscattered electron (BSE) images were simultaneously obtained during the mapping. Raw data of X-ray elemental maps, SE, and BSE were imported and processed for brightness and contrast by ImageJ^41^. A red–green–blue color scheme in ImageJ was applied to make a false color map.

### Tardigrade treatment

*Dactylobiotus* sp., a member of the Eutardigrada was collected near the King Sejong Station, at the King George Island, Antarctica during the 2014–2015 field season by a KOPRI ecology team (led by Dr. Sanghee Kim). Specimens were killed by hot water^42^, and fixed in glycerol^43^ with mounting medium on microscope slides with 18 × 18 mm square coverslips^44^. DIC images were taken with an AxioCam HRc camera mounted on a Zeiss Imager A2 microscope. For SEM images, samples were fixed in 5% formaldehyde overnight, heated in a double boiler at ~70–80 ℃, and washed with distilled water three times. Subsequently, dehydration was performed twice in series with ethanol of 50%, 60%, 70%, 80%, 90%, and 100%. Then, the samples were critical point dried and sputter coated with gold. The SEM images were acquired using a JEOL JSM-6610 SEM. The samples are deposited in the Korea Polar Research Institute, prefixed with KOPRIF (KOPRIF50001, 50002).

### Data availability

The data that support the findings of this study are available from the corresponding authors by bona fide request.

## Electronic supplementary material

Supplementary Information
