# Supplementary material for: Brain and eyes of Kerygmachela reveal protocerebral ancestry of the panarthropod head
Source: Nat Commun. 2018 Mar 9;9:1019. doi: 10.1038/s41467-018-03464-w (PMC5844904; doi:10.1038/s41467-018-03464-w)
Supplement: Supplementary file 1 — Supplementary Information [file 41467_2018_3464_MOESM1_ESM.pdf]

Supplementary Information

Brain and eyes of *Kerygmachela* reveal protocerebral ancestry of the panarthropod head

Park et al.

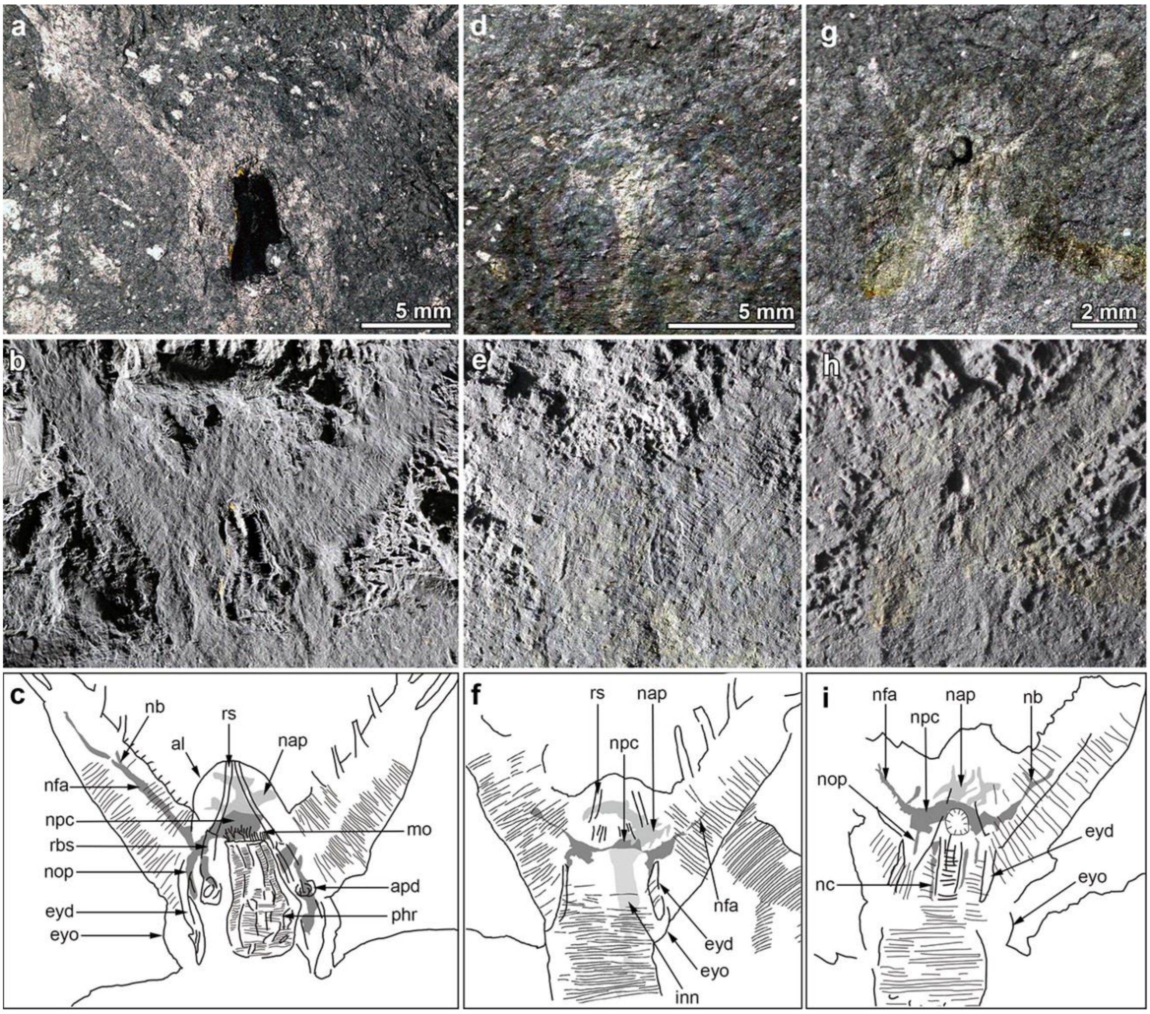

Supplementary Figure. 1. ***Kerygmachela kierkegaardi.*** **a-c,** MGUH 32051. **a,** under high-angle polarized lighting. **b,** under low-angle lighting. **c,** interpretive drawing of **a** and **b**. **d–f,** MGUH 32052. **d,** under high-angle polarized lighting. **e,** under low-angle lighting. **f,** interpretive drawing of **d** and **e**. **g–i,** MGUH 32053. **g,** under high-angle polarized lighting. **h,** under low-angle lighting. **i,** interpretive drawing of **g** and **h**. al, anterior lobe; apd; apodeme; eyd, displaced eye structure; eyo, original outline of eye lobe; inn, non-neural impression; mo, mouth opening; nap, anterior neural projection; nb, branching of nerve; nc, nerve cord; nfa, frontal appendage nervous tract; npc, protocerebrum; nop, optic nerve; phr, pharynx; rbs, basal sheath for rostral spine; rs, rostral spine.

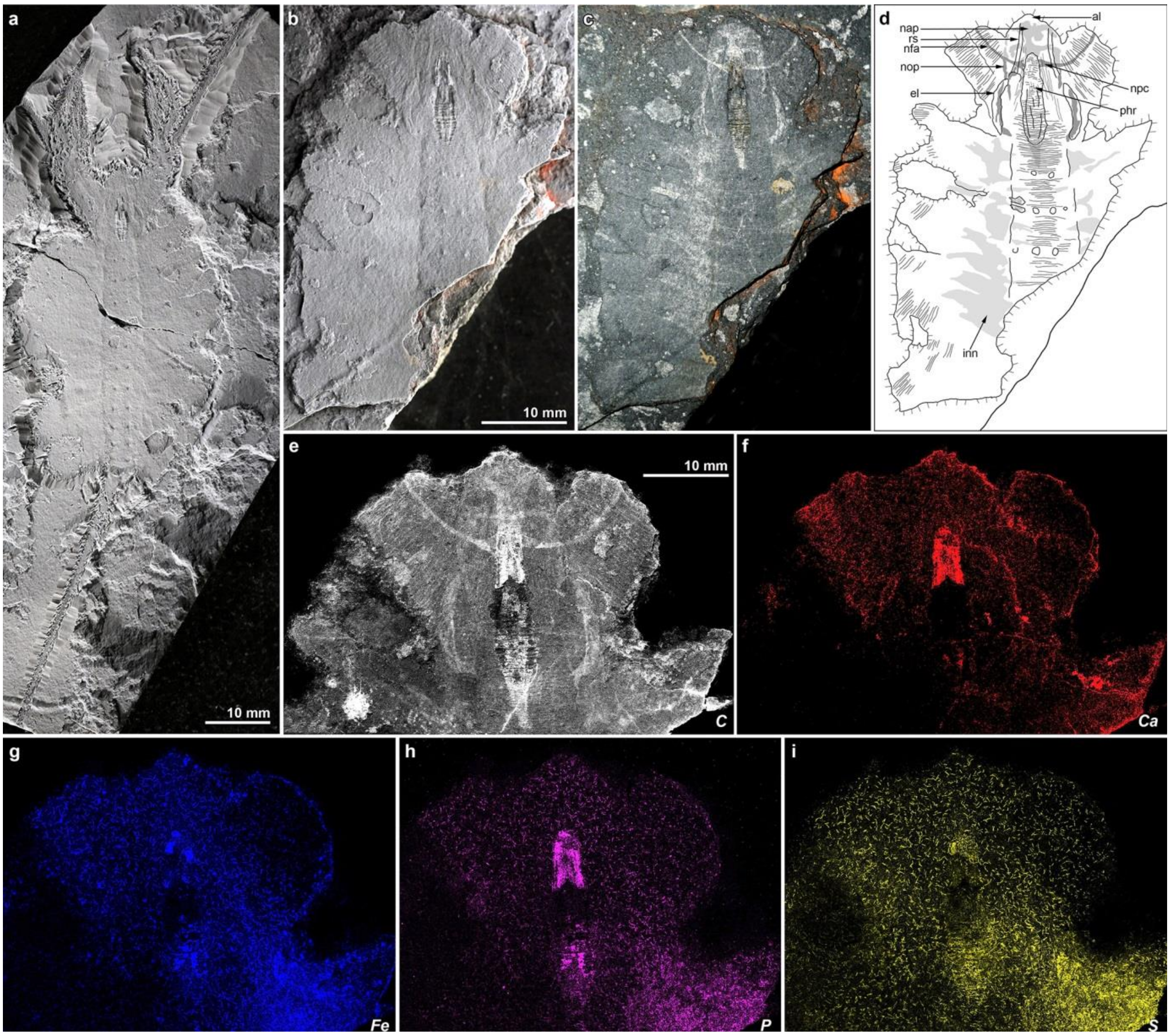

Supplementary Figure 2. ***Kerygmachela kierkegaardi*****. a,** MGUH 32048a, white-coated specimen. **b–i,** MGUH 32048b. **b,** polynomial texture mapping image. **c,** under high-angle polarized lighting. **d,** interpretive drawing of **b** and **c**. **e–i,** Wavelength Dispersive X-ray elemental maps of the head region. **e,** carbon map. **f,** calcium map. **g,** iron map. **h,** phosphate map. **i,** sulphur map. al, anterior lobe; el, eye lobe; inn, non-neural impression; nap, anterior neural projection; nfa, frontal appendage nervous tract; npc, protocerebrum; nop, optic neuron; phr, pharynx; rs, rostral spine.

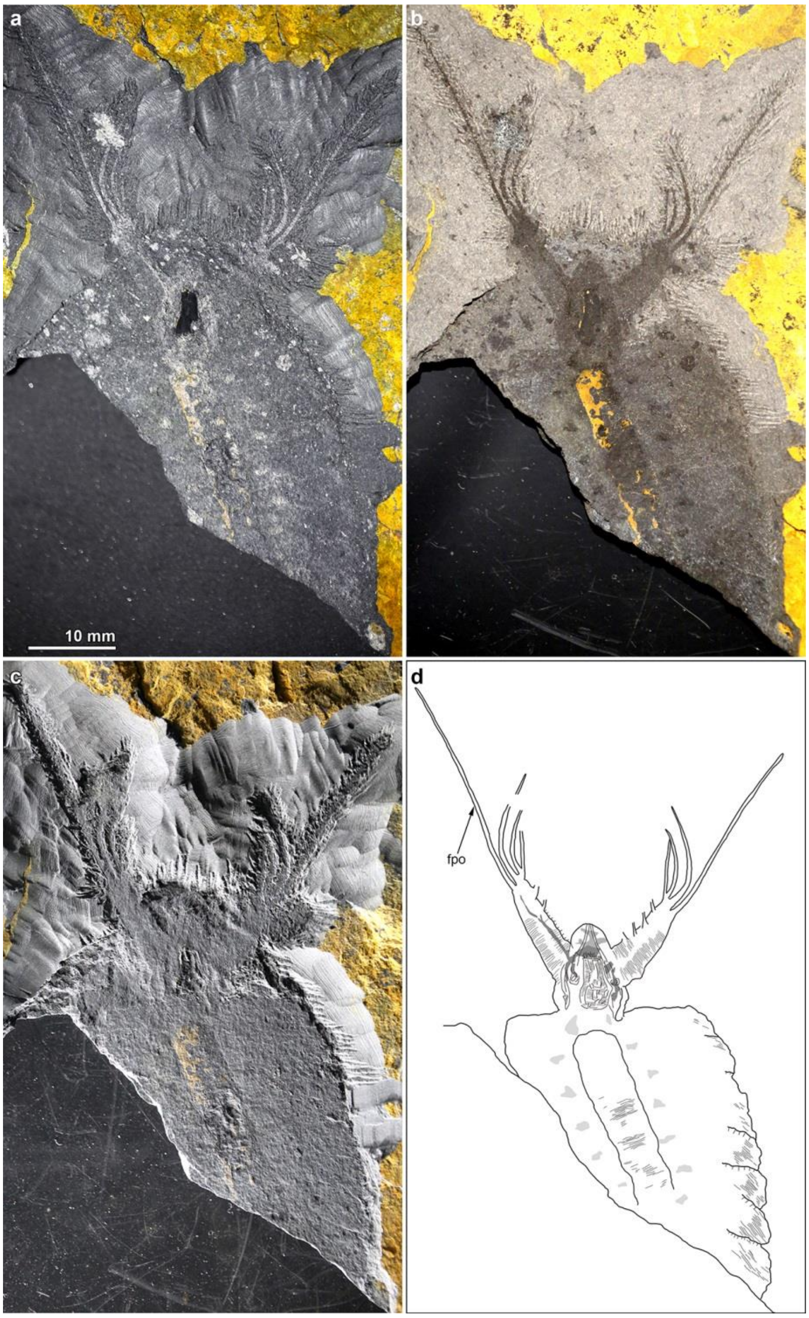

Supplementary Figure 3. ***Kerygmachela kierkegaardi*****.** MGUH 32054. **a,** under high-angle polarized lighting. **b,** under high-angle polarized lighting with crossed nicols. **c,** under low-angle lighting. **d,** interpretive drawing of **a** and **c**. fpo, outermost frontal process.

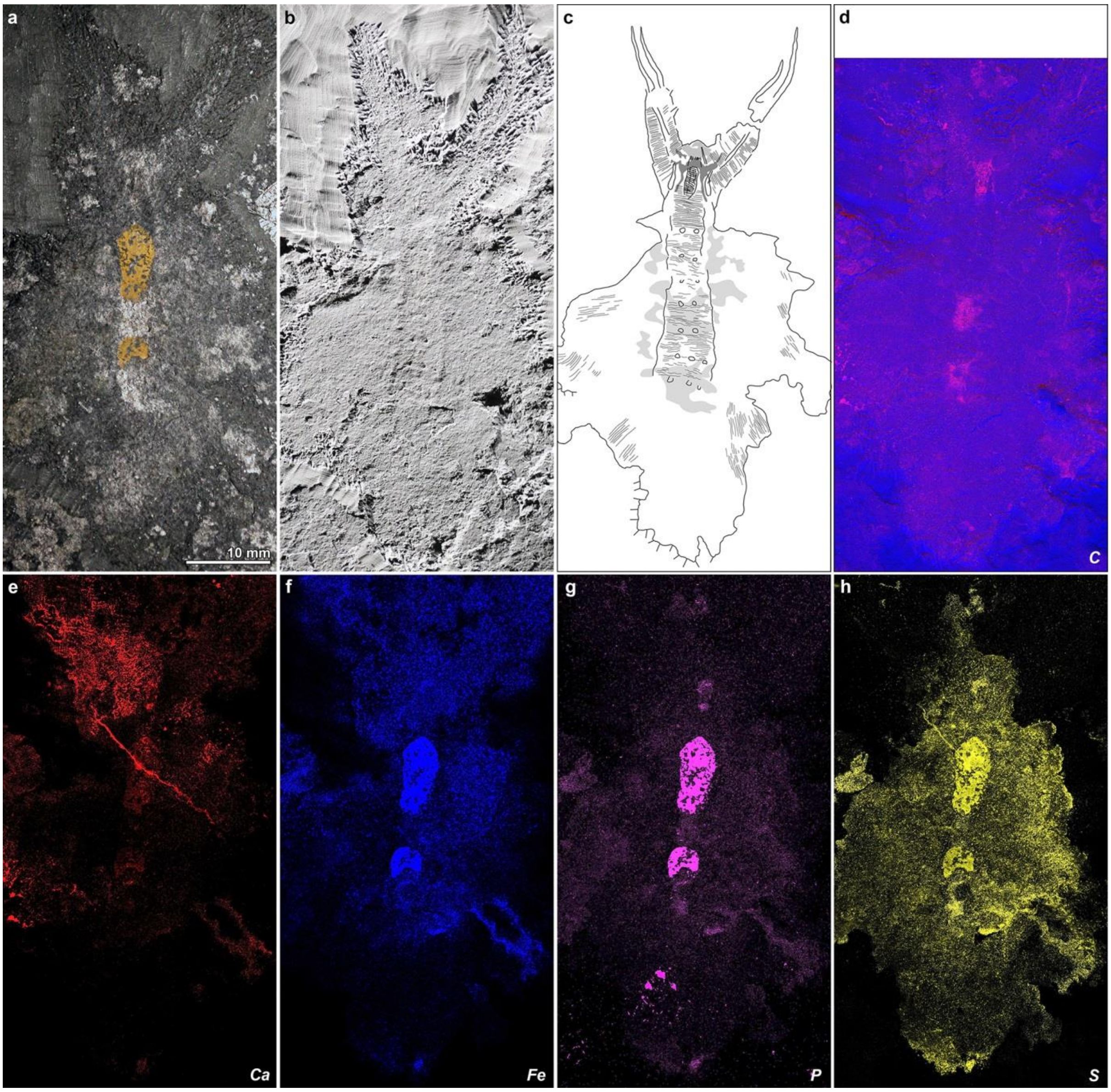

Supplementary Figure 4. ***Kerygmachela kierkegaardi*****.** MGUH 32050. **a,** under high-angle polarized lighting. **b,** white-coated specimen. **c,** interpretive drawing of **a** and **b**. **d–h,** Wavelength Dispersive X-ray elemental maps. **d,** carbon-rich region (red), superimposed upon the topographic map (blue). **e,** calcium map. **f,** iron map. **g,** phosphate map. **h,** sulphur map.

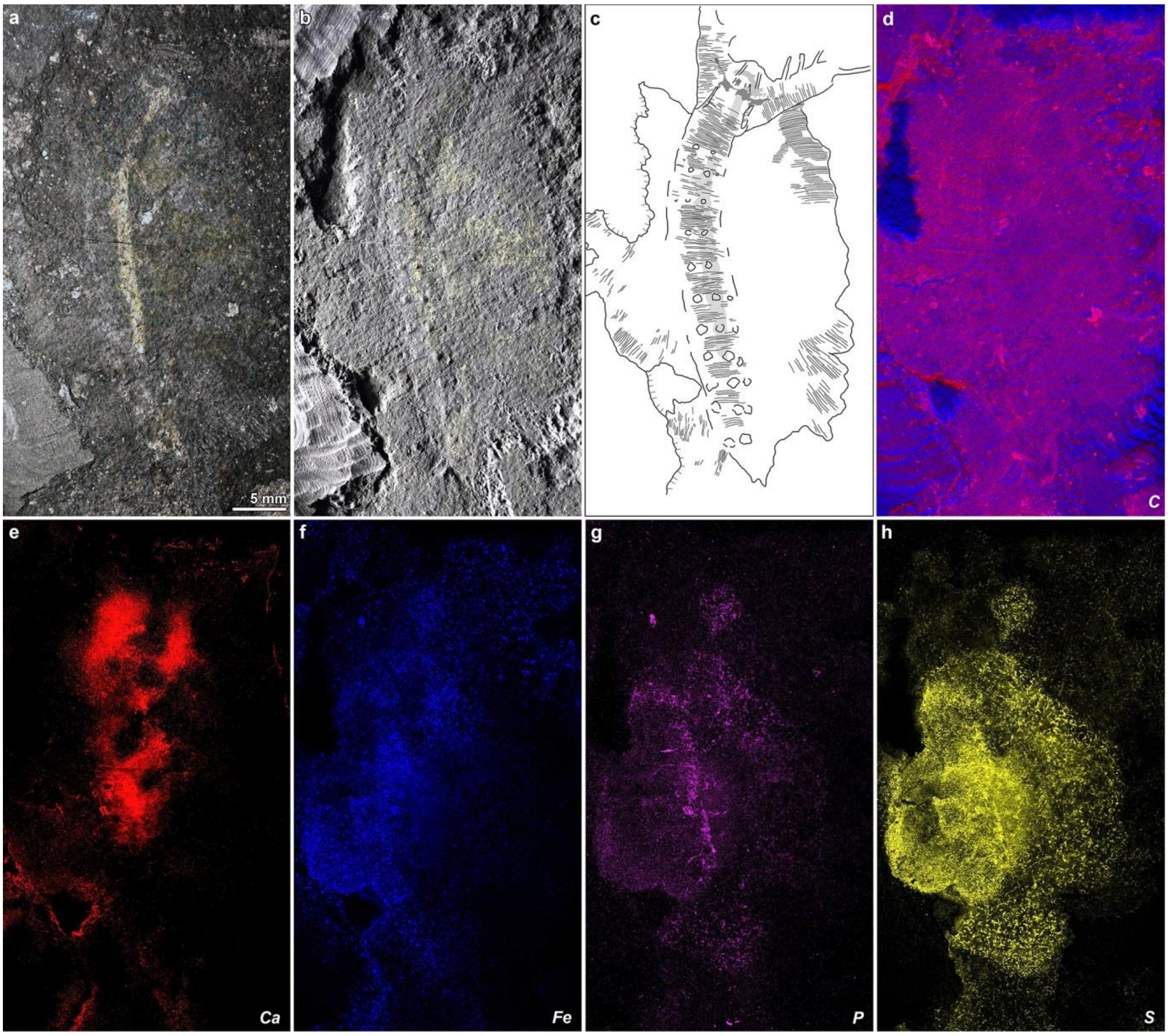

Supplementary Figure 5. ***Kerygmachela kierkegaardi*****.** MGUH 32052. **a,** under high-angle polarized lighting. **b,** under low-angle lighting. **c,** interpretive drawing of **a** and **b**. **d–h,** Wavelength Dispersive X-ray elemental maps. **d,** carbon-rich region (red), superimposed upon the topographic map (blue). **e,** calcium map. **f,** iron map. **g,** phosphate map. **h,** sulphur map.

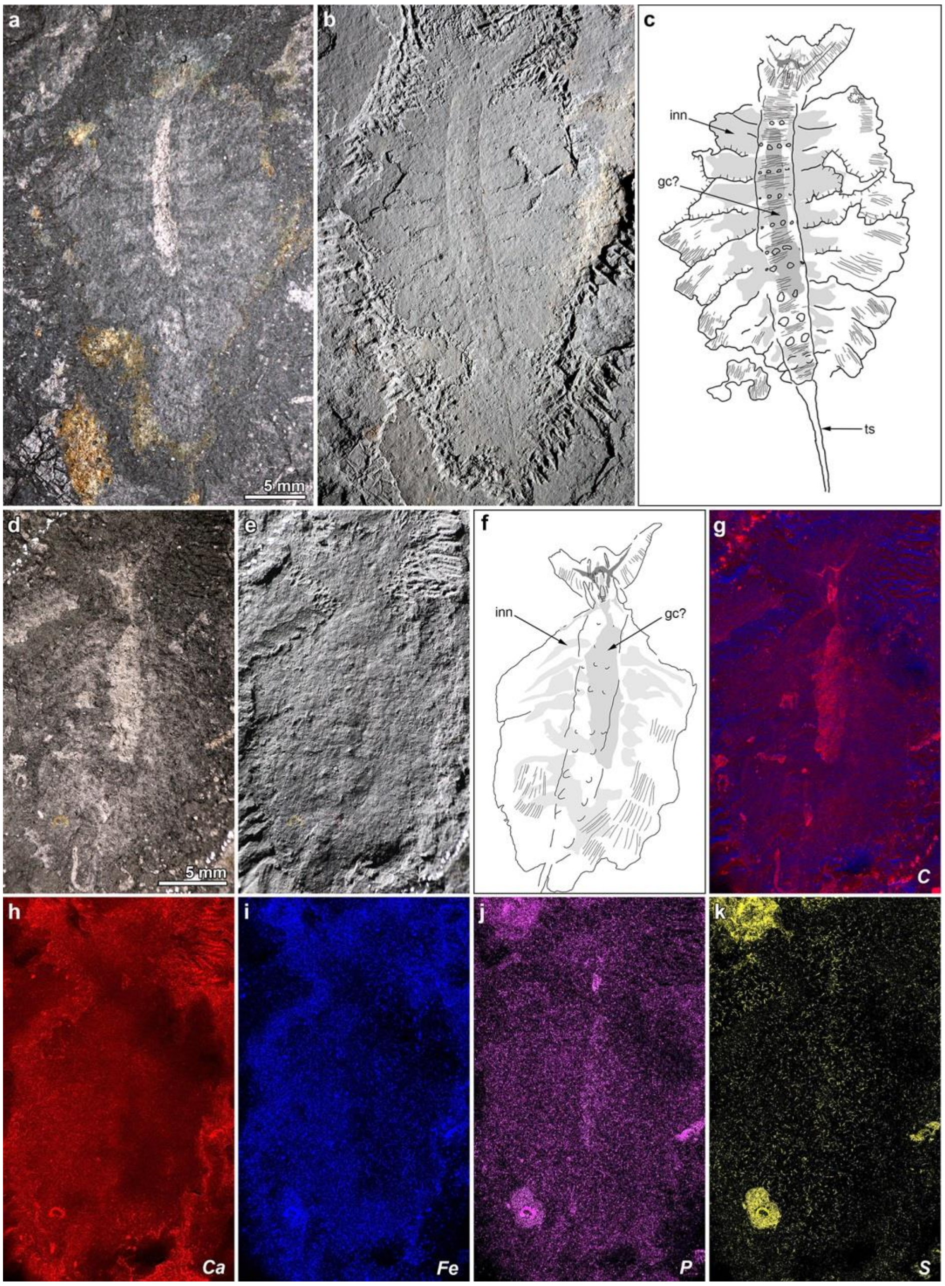

Supplementary Figure 6. ***Kerygmachela kierkegaardi*****. a–c,** MGUH 32053. **a,** under high-angle polarized lighting. **b,** white-coated specimen. **c,** interpretive drawing of **a** and **b**. **d–k,** MGUH 32049. **d,** under high-angle polarized lighting. **e,** under low-angle lighting. **f,** interpretive drawing of **d** and **e**. **g–k,** Wavelength Dispersive X-ray elemental maps. **g,** carbon-rich region (red), superimposed upon the topographic map (blue). **h,** calcium map. **i,** iron map. **j,** phosphate map. **k,** sulphur map. gc, gut contents; inn, non-neural impression; ts, tail spine.

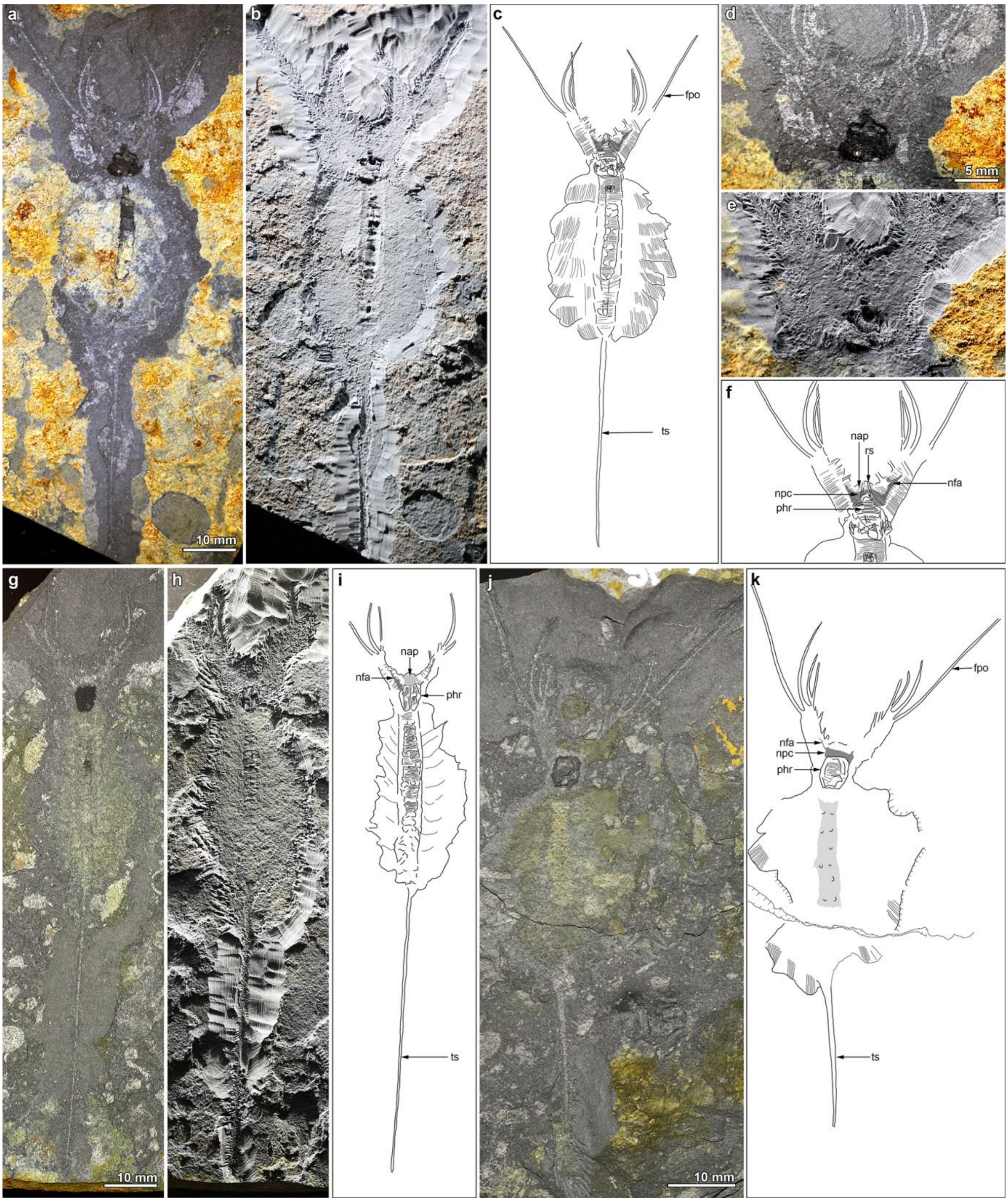

Supplementary Figure 7. ***Kerygmachela kierkegaardi*****. a–f,** MGUH 32055. **a,** under high-angle polarized lighting. **b,** white-coated specimen. **c,** interpretive drawing of **a** and **b**. **d–f,** head region. **d,** under high-angle polarized lighting. **e,** under low-angle lighting. **f,** interpretive drawing of **d** and **e**. **g–i,** MGUH 32056. **g,** under high-angle polarized lighting. **h,** white-coated specimen. **i,** interpretive drawing of **g** and **h**. **j–k,** MGUH 32057. **j,** under high-angle polarized lighting. **k,** interpretive drawing of **j**. fpo, outermost frontal process; nap, anterior neural projection; nfa, frontal appendage nervous tract; npc, protocerebrum; phr, pharynx; rs, rostral spine; ts, tail spine.

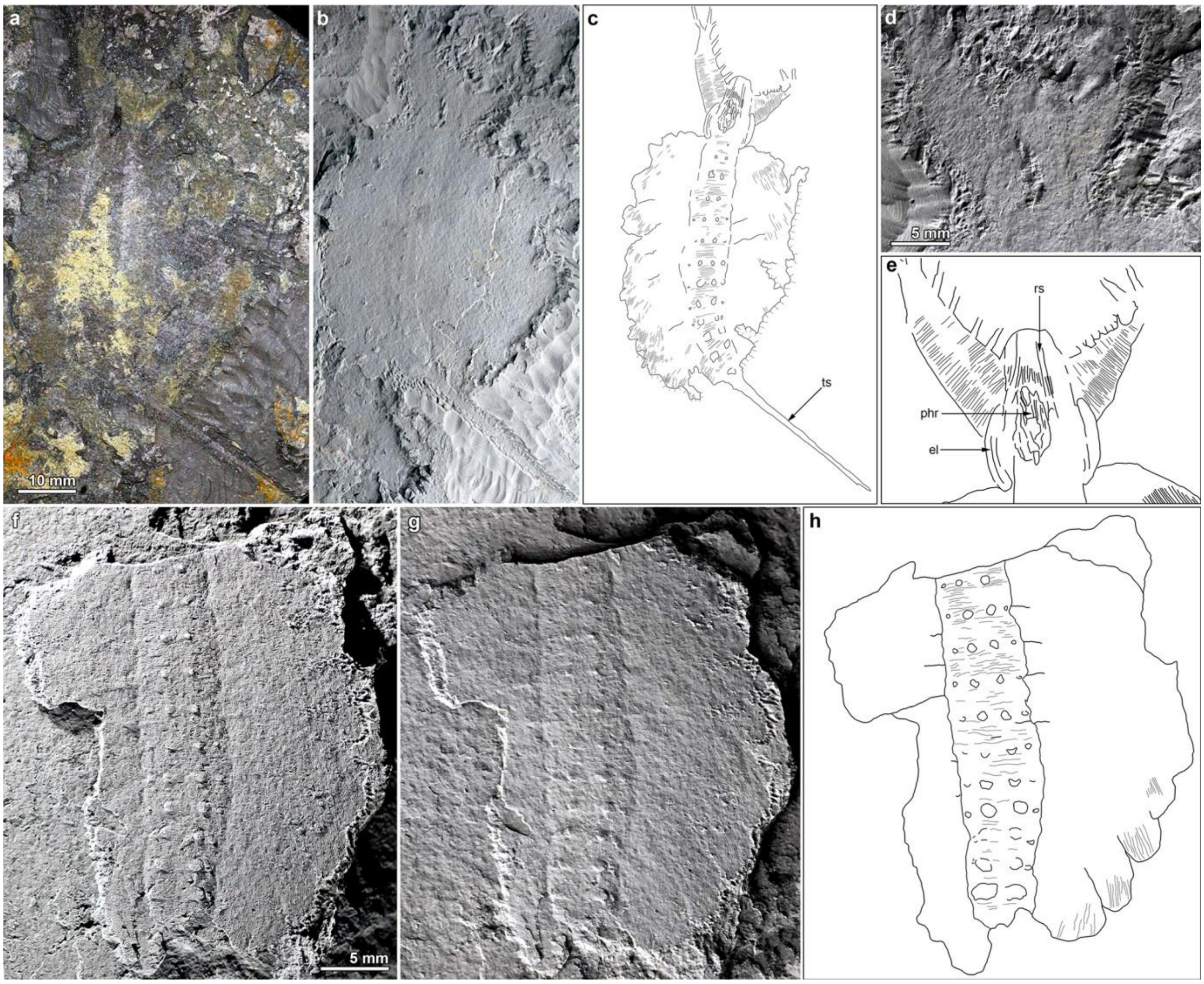

Supplementary Figure 8. ***Kerygmachela kierkegaardi*****. a–e,** MGUH 32058. **a,** under high-angle polarized lighting. **b,** polynomial texture mapping image. **c,** interpretive drawing of **b**. **d, e,** head region. **d,** under low-angle lighting. **e,** interpretive drawing of **d**. **f–h,** MGUH 32059. **f,** white-coated specimen. **g,** polynomial texture mapping image. **h,** interpretive drawing of **g**. el, eye lobe; phr, pharynx; rs, rostral spine; ts, tail spine.

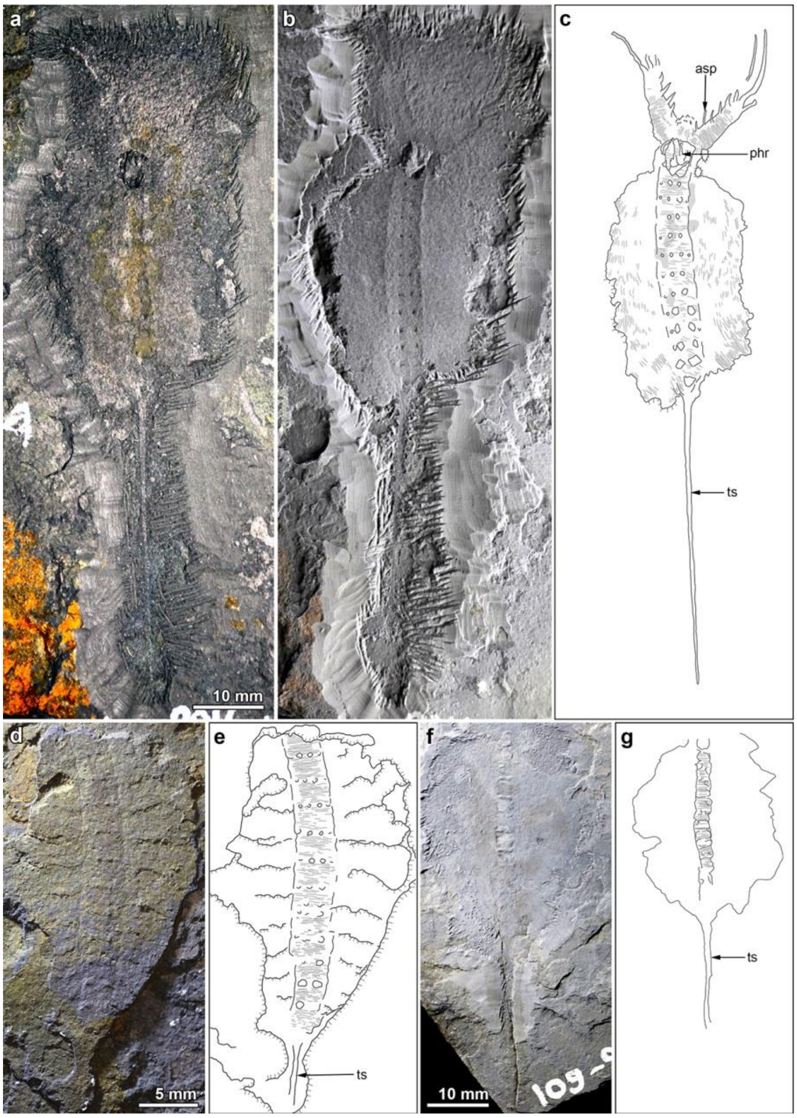

Supplementary Figure 9. ***Kerygmachela kierkegaardi*****. a–c,** MGUH 32060. **a,** under high-angle polarized lighting. **b,** polynomial texture mapping image. **c,** interpretive drawing of **b**. **d, e,** MGUH 32061. **d,** under low-angle lighting. **e,** interpretive drawing of **d**. **f, g,** MGUH 32062. **f,** white-coated specimen. **g,** interpretive drawing of **f**. asp, anterior spine; el, eye lobe; phr, pharynx; rs, rostral spine; ts, tail spine.

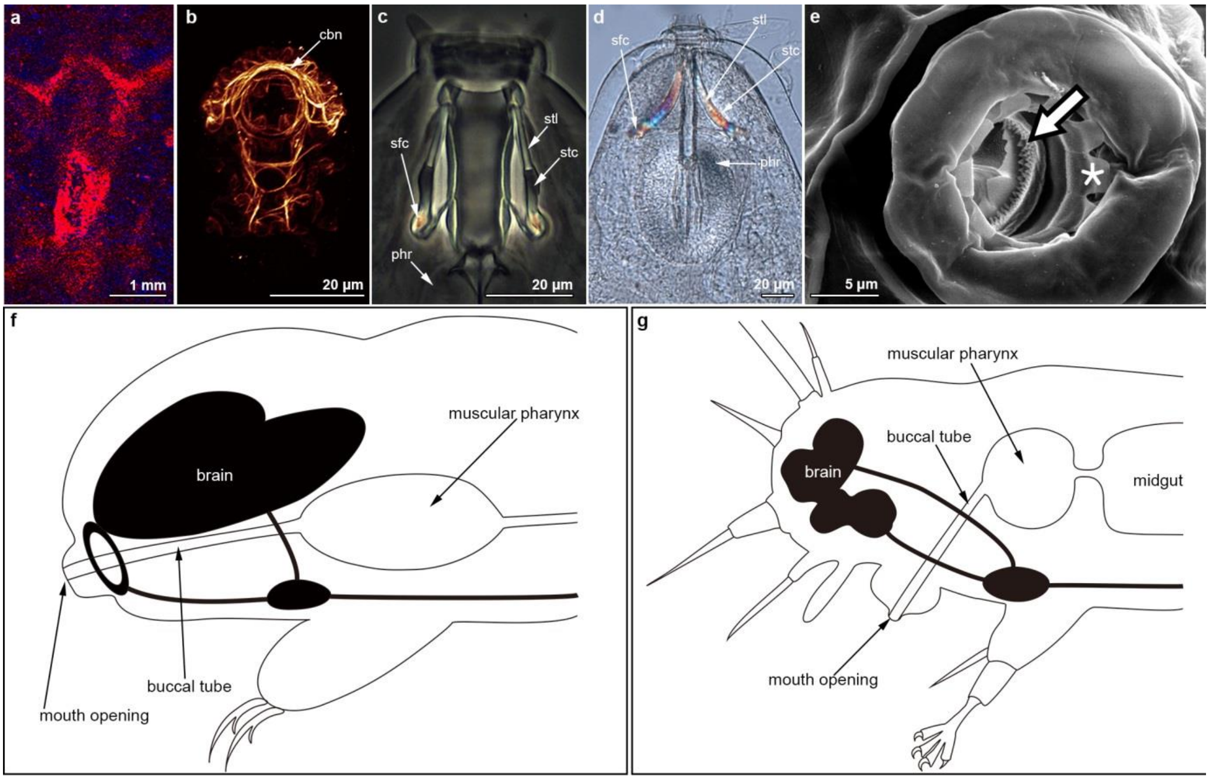

Supplementary Figure 10. Wavelength Dispersive X-ray elemental map of *Kerygmachela kierkegaardi* and detailed features of tardigrades. a, Wavelength Dispersive X-ray elemental map of carbon of the head region of MGUH 32049, showing the anteriorly-rounded central brain neuropil. Carbon-rich region (red) is superimposed upon the topographic map (blue). **b,** Confocal laser scanning microscopic image of the central nervous system of the eutardigrade *Hypsibius dujardini* which was labeled for acetylated α-tubulin. Image courtesy of V. Gross. **c,** Bucco-pharyngeal apparatus of the eutardigrade *Milnesium berladnicorum*[1]. Image courtesy of D. A. Ciobanu. **d,** Differential Interference Contrast (DIC) image of the bucco-pharyngeal apparatus of the eutardigrade *Dactylobiotus* sp., KOPRIF5001. **e,** SEM image of the mouth part of *Dactylobitus* sp. Peribuccal lamellae (asterisk) and peribuccal lamellae (arrow), KOPRIF5002. **f,** Schematic figure of nervous system and buccopharyngeal apparatus of the eutardigrades *Macrobiotus* cf. *harmsworth* (modified from 2). **g,** schematic figure of nervous system and buccopharyngeal apparatus of the heterotardigrade *Actinarctus doryphorus*[3]. The third brain lobes (bl3) and subpharyngeal ganglion (g0) as originally identified by ref (3) has been removed, as these have not been confirmed by other studies[4–7]. cbn, central brain neuropil; phr, pharynx; sfc, stylet furca; stl, stylet; stc, stylet coat.

Supplementary References

1. Ciobanu, D. A., Zawierucha, K., Moglan, I. & Kaczmarek, Ł. *Milnesium berladnicorum* sp. n. (Eutardigrada, Apochela, Milnessidae), a new species of water bear from Romania. *Zookeys* **429,** 1–11 (2014).

2. Ortega-Hernández, J. Homology of head sclerites in Burgess shale euarthropods. *Curr. Biol.* **25,** 1–7 (2015).

3. Persson, D. K., Halberg, K. A., Jørgensen, A., Møbjerg, N., Kristensen, R. M. Brain anatomy of the marine tardigrade *Actinarctus doryphorus* (Arthrotardigrada). *J. Morphol.* **275,** 173–190 (2014).

4, Persson, D. K., Halberg, K. A., Jørgensen, A., Møbjerg, N., Kristensen, R. M. Neuroanatomy of *Halobiotus crispae* (Eutardigrada: Hypsibiidae): Tardigrade brain structure supports the clade Panarthropoda. *J. Morphol*. **273**, 1227–1235 (2012).

5, Schulze, C., Neves, R. C., Schmidt-Rhaesa, A. Comparative immunohistochemical investigation on the nervous system of two species of Arthrotardigrada (Heterotardigrada, Tardigrada). *Zool. Anz*. **253**, 225–235 (2014).

6. Mayer, G., Martin, C., Rüdiger, J., Kauschke, S., Stevenson, P. A., Poprawa, I., Hohberg, K., Schill, R. O., Pflüger, H.-J., Schlegel, M. Selective neuronal staining in tardigrades and onychophorans provides insights into the evolution of segmental ganglia in panarthropods. *BMC Evol. Biol*. **13**, 230 (2013).

7. Zantke, J., Wolff, C., Scholtz, G. Three-dimensional reconstruction of the central nervous system of *Macrobiotus hufelandi* (Eutardigrada, Parachela): implications for the phylogenetic postion of Tardigrada. *Zoomorphology*. **127**, 21–36 (2008).
